# Application of Ancestry Informative Markers to Association Studies in European Americans

**DOI:** 10.1371/journal.pgen.0040005

**Published:** 2008-01-18

**Authors:** Michael F Seldin, Alkes L Price

**Affiliations:** University of Chicago, United States of America

Recently, whole genome association (WGA) studies have accelerated progress in the search for genetic variations underlying the inheritance of complex genetic diseases. Although population differences in allele frequencies are usually small, these studies have demonstrated the importance of accounting for population differences in order to reduce false positive associations. Even within a continental population, population stratification—ancestry differences between cases and controls—can cause false associations at markers whose frequency differs across subpopulations [1,2]. For example, in a recent WGA study of rheumatoid arthritis in European Americans, markers in the *LCT* and *IRF4* genes would have been falsely implicated as associated to disease without the application of methods to control for stratification [3]. Similar empirical examples of population stratification exist for other phenotypes, and genetic risk has been reported to vary across Europe for a wide range of diseases [4–8]. In general, investigators should be alerted to consider population stratification when WGA data indicates that a particular marker shows a strong frequency gradient across Europe.

Methods have already been developed to control for population stratification in the initial stage of WGA studies, in which data from hundreds of thousands of markers is generated [9,10]. However, controlling for stratification is just as important in replication studies in independent sample sets, which will focus on a small number of markers. Similarly, candidate gene studies and fine mapping or sequencing studies will also require attention to population differences. Because a small number of candidate markers will not be sufficiently informative for ancestry, and genotyping a large number of markers is expensive, there is a need for small panels of ancestry informative markers (AIMs) that can be used to accurately infer ancestry [11]. We focus here on European Americans, a structured population that is often sampled in association studies.

Multiple studies have shown that the largest source of population structure in European Americans involves a north–south (or northwest–southeast) cline through Europe [9,12,13]. However, subtler effects involving other regional or ethnic differences can also contribute to stratification. An important question is which ancestries should be evaluated in replication studies by genotyping of AIMs at additional cost. The answer to this question will vary from study to study, depending on factors such as the collection location of cases and controls, the phenotype being studied, and considerations of cost. For example, a study of a phenotype with known ancestry differences, in which cases are collected from a large city and controls are collected from throughout the country, would be well-advised to define ancestry to the fullest extent possible. On the other hand, a study of a phenotype with no known ancestry differences, involving cases and controls rigorously matched by location, might choose to bypass the use of AIMs entirely. An intermediate option would be to model only north–south ancestry, addressing the single most likely source of stratification at partial cost, with some residual risk of stratification.

Two research papers by our two groups in the current issue of *PLoS Genetics* provide a broad assessment of European American population structure, and also provide several sets of AIMs for inferring ancestry in European Americans [3,4]. Our respective sets of AIMs were ascertained using different pairs of populations, but have each been shown to be effective in discerning the ancestries for which they were ascertained. The Price et al. study analyzes WGA data from the Affymetrix 500 K and Illumina 300 K platforms and describes a set of 100 AIMs ascertained using northwest versus southeast European ancestry (Price100) and a set of 200 AIMs ascertained using southeast European versus Ashkenazi Jewish ancestry (Price200) [4]. The Tian et al. study analyzes WGA data from the Illumina 300 K and 500 K platforms and describes a set of 192 AIMs ascertained using northern European versus Ashkenazi Jewish ancestry (Tian192) and a set of 1,211 AIMs ascertained using Irish versus other northern European ancestry (Tian1211) [3]. It should be stressed that combined information from either a very large set of markers or a set of highly specialized markers is required to distinguish the ancestries of these genetically very similar populations, whose real or perceived group differences may often be dominated by environmental, social, and cultural factors. Below, we outline the possible choices of marker sets for inferring various ancestries. In each case, a method such as structured association or principal components analysis can be applied to genotype data to correct for stratification.

To correct for stratification along the north–south (or northwest–southeast) cline, either the Price100 or Tian192 marker sets can be used. (The Tian192 markers, which were ascertained using northern European versus Ashkenazi Jewish ancestry, are effective in distinguishing north–south ancestry because southern Europeans attain intermediate ancestry values as compared to values at one extreme for northern Europeans.) To correct for stratification involving both north–south and Ashkenazi Jewish ancestry, one option is to use the Price100+Price200 marker sets, which together separate north, south, and Ashkenazi ancestry into three distinct clusters. Another option is to use the Tian192 marker set, which models these three ancestries along a single axis and will be sufficient in the case that the phenotype being analyzed has intermediate values for southern European as compared to northern European versus Ashkenazi Jewish ancestry. Finally, to correct for stratification involving a west–east gradient within northern Europe (e.g., Irish versus other northern European ancestry), the Tian1211 marker set is the only set of AIMs available.

We note that the initial information from a WGA study can help to determine the appropriate choice of AIMs for a replication study. An important question is, are there ancestry differences between cases and controls in the initial WGA study—and if so, which ancestries contribute to this effect, and do sets of AIMs correct for stratification in the WGA data as effectively as the complete set of WGA markers? Of course, a caveat to such an approach is the requirement that the cases and controls used for replication are demographically matched to those used in the initial study.

It is also worth noting that for some studies the analysis of population structure might precede WGA. Thus, depending on the number of case and control samples and the cost of prescreening with AIM panels, it may be advantageous to first match cases and controls for ancestry. This could improve the power of the study, for two reasons. First, methods to correct for stratification in a scenario with poorly matched cases and controls will lead to an inevitable loss of power in a WGA study. Second, if a variant is more polymorphic or has higher relative risk in samples of a particular ancestry, then a more genetically homogeneous group of subjects (for example, focusing on Ashkenazi Jewish ancestry [14]) may be more likely to reveal that variant.

We caution that population stratification is not the only source of false positive associations in disease studies. In particular, differences in DNA quality or laboratory treatment between cases or controls may produce spurious signals that will not be addressed by using AIMs [15]. Subtle instances of differential bias will be difficult to detect in studies involving a small number of markers, but a possible diagnostic check is to compare rates of missing data between cases and controls.

In conclusion, replication and candidate gene studies in European Americans can now make use of AIMs for examining north–south, Ashkenazi Jewish, and Irish ancestry. Though European Americans could exhibit additional even subtler population structure effects, these would contribute much less strongly to stratification and would require a higher number of markers to discern, limiting their relevance to AIM sets. Going forward, the widespread implementation of AIMs may benefit from specialized products on dedicated platforms to reduce costs. Discussions are currently under way to achieve this, and we anticipate that specialized AIM products will be commercially available to the research community in the near future. We also envision that the increasing explosion of WGA data will aid the ascertainment of AIM panels for a broader range of populations, beyond European Americans.
